# A Case of Diffuse Metastatic Melanoma With Massive Spontaneous Hemoperitoneum

**DOI:** 10.7759/cureus.8555

**Published:** 2020-06-10

**Authors:** Jamin K Addae, Robert D Rawlinson, Abigail Adjei, Bruce Brenner, Karin Blumofe

**Affiliations:** 1 General Surgery, Charles E. Schmidt College of Medicine, Florida Atlantic University, Boca Raton, USA; 2 Surgery, Charles E. Schmidt College of Medicine, Florida Atlantic University, Boca Raton, USA; 3 Epidemiology and Public Health, Health Science Center at Houston, University of Texas, Austin, USA

**Keywords:** melanoma, hemoperitoneum, diffuse metastatic melanoma, skin cancer, occult melanoma

## Abstract

The incidence rate for melanoma continues to rise in the USA. The majority of melanoma cases are detected at an early stage and are amenable to surgical excision. Advanced melanoma with diffuse intraabdominal metastasis is rare. We present a case of a 50-year-old female with no known primary or history of melanoma who presented with massive intraabdominal bleeding secondary to diffuse metastatic melanoma with peritoneal implants. Diagnosing metastatic melanoma could be challenging. Clinicians should be aware of hemoperitoneum or peritoneal carcinomatosis as potential manifestations of malignant melanoma to expedite appropriate management.

## Introduction

Melanoma is the fifth most common cause of cancer in both men and women in the USA [1]. The majority of cases are diagnosed at an early stage where surgical excision alone is often curative [2, 3]. Advanced cases may require immunotherapy, targeted therapy, chemotherapy, or radiation therapy in addition to surgical excision [4].

Cutaneous melanoma may regress spontaneously without any surgical intervention. Metastatic disease can be challenging to diagnose in these cases when there is no known primary [5]. Patients with mucosal or ocular melanoma may also present with metastases prior to developing symptoms from the primary tumor. We present a rare case of a patient with massive hemoperitoneum as a complication of peritoneal deposits of melanoma without a known primary.

## Case presentation

A 50-year-old woman presented with a four-day history of the left flank and lower abdominal pain. Her pain worsened with positional changes and waxed and waned. She reported nausea and abdominal distension, but no emesis, fever, or chills. She denied any urinary symptoms, hematemesis, bleeding per rectum, or use of any anticoagulants. Her past medical history was unremarkable. Her last Papanicolau (Pap) smear was nine years ago and was normal. Her past surgical history was significant for rhinoplasty and breast augmentation.

On admission, she was tachycardic, but not febrile or hypotensive. On physical examination, she was found to be pale with a distended abdomen and generalized abdominal tenderness but no peritoneal signs.

Her initial Hgb was 6.6g/dl and subsequently dropped to 5.3g/dl three hours later. She had a normal coagulation profile. Her lactate was 2.0 mmol/L, and lactate dehydrogenase was 1440U/L.

**Figure 1:**
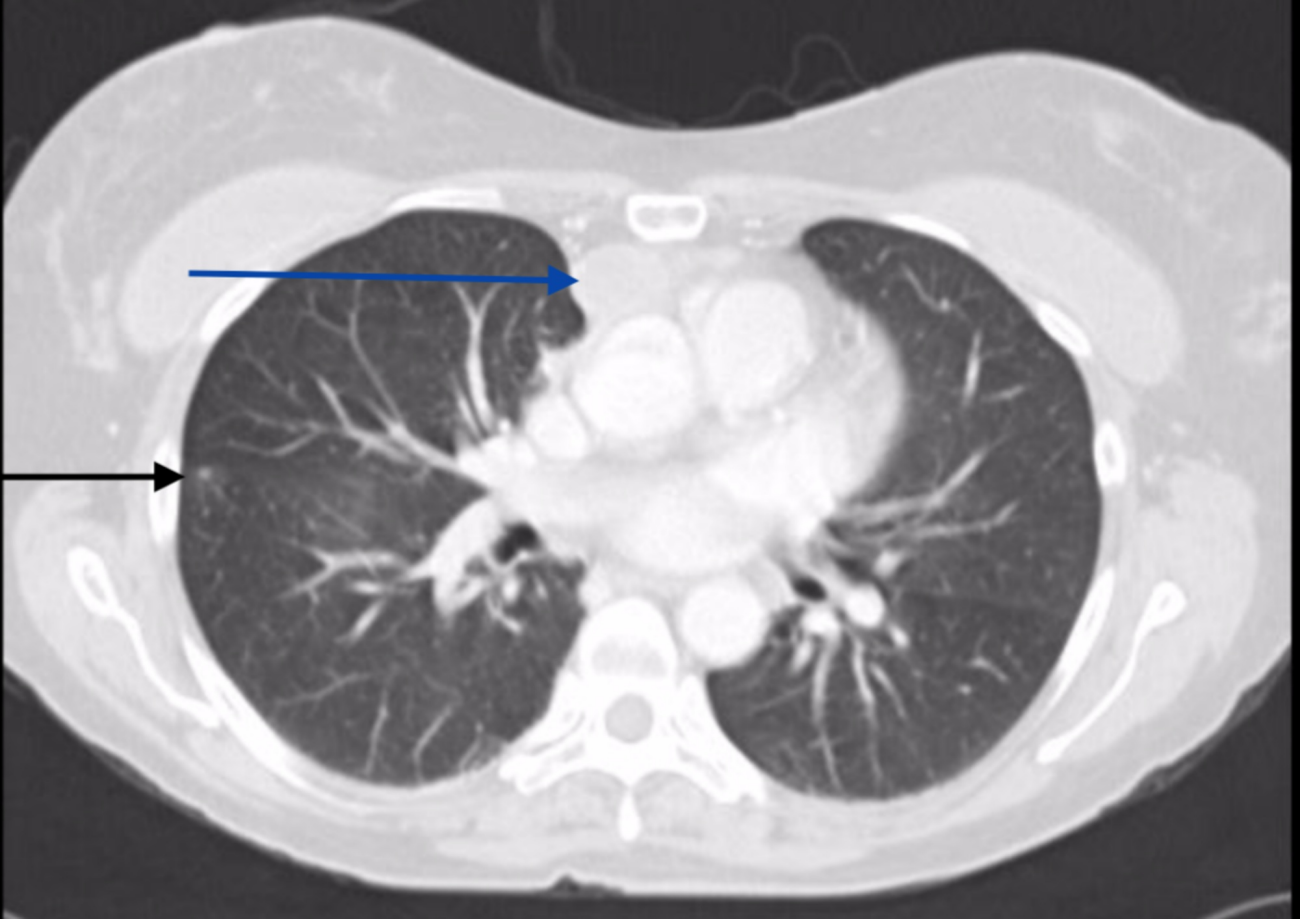
Axial chest CT showing right lung lesion Axial chest CT showing right lung lesion (black arrow) and anterior mediastinal mass (blue arrow).

**Figure 2:**
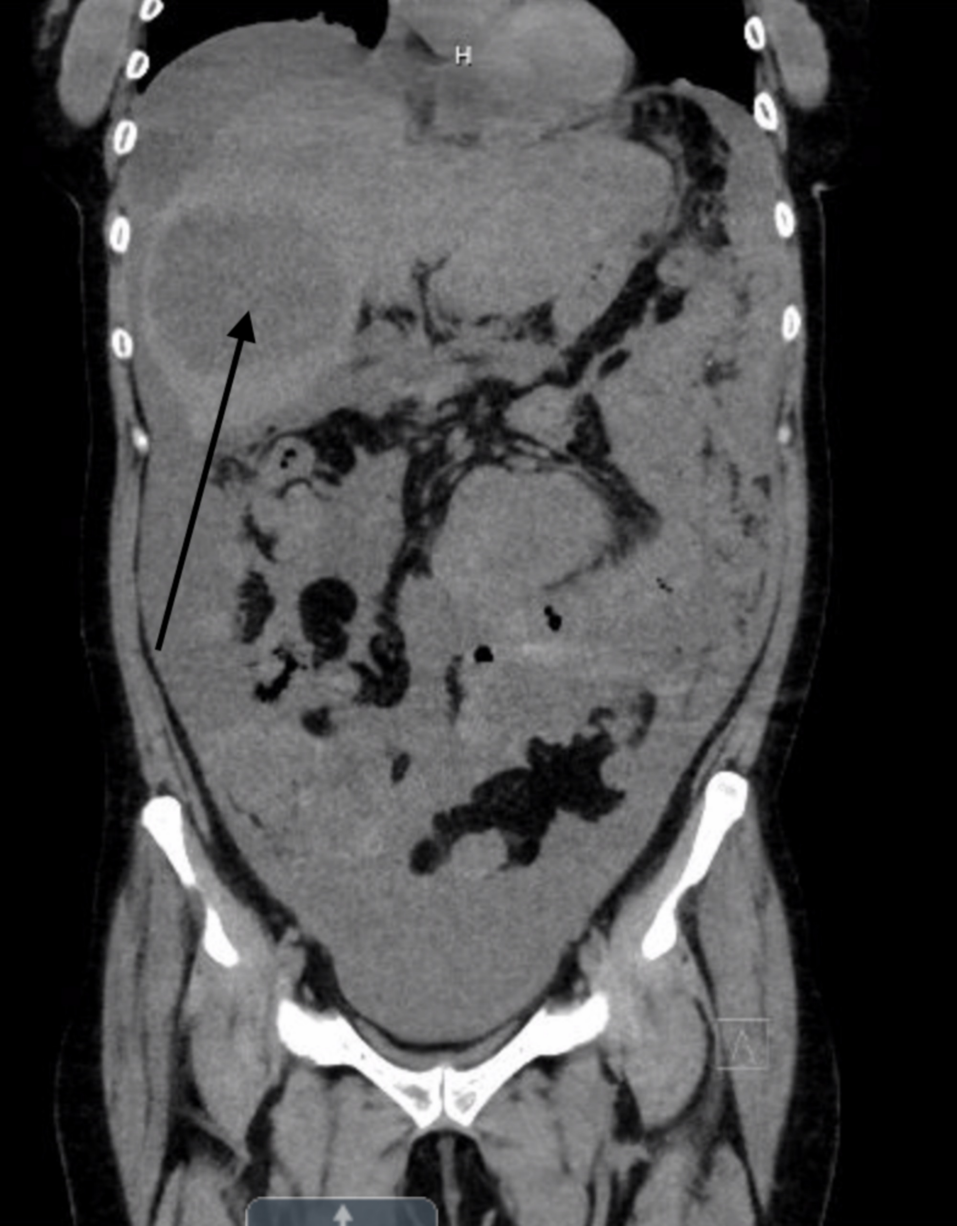
Coronal view of CT chest and abdomen Coronal view of CT chest and abdomen showing a large right hepatic lobe mass (black arrow) measuring 8.7 cm x 8.7 cm and fluid in the right upper quadrant.

Computed tomography of the chest, abdomen, and pelvis showed bilateral small nodular lung masses up to 4 mm, a 5.2 cm x 4.2 cm x 2.1 cm mediastinal mass near the thymus, a right hepatic lobe mass measuring 8.7 cm x 8.7 cm, fluid in the right upper quadrant, the hyperdense lesion in the left kidney measuring 5.6 cm, right adrenal nodule measuring 2.6 cm and scattered enlarged peripancreatic and periportal lymph nodes. There was a large amount of free fluid in the pelvis, multiple omental deposits, and a heterogenous lesion on T11 vertebral body suspicious for metastatic disease (Figures 1 and 2).

Tumor markers, including alpha-fetoprotein (AFP), carbohydrate antigen 19-9 (CA 19-9), and carcinoembryonic antigen (CEA) were normal, with an elevated cancer antigen 125 (CA 125) of 186.4 (normal < 35U/ml), stool occult blood was negative.

The patient was transfused two units of packed red blood cells on admission and as needed to keep her Hgb above 8g/dl. She underwent ultrasound-guided paracentesis of 2.5L of dark hemorrhagic fluid from the abdomen.

Computed tomographic angiography was obtained with the aim to identify the source of the bleed. No active extravasation was seen. Given the ongoing drop in her hemoglobin despite four units of packed red blood cells (PRBC) transfusion and persistent tachycardia, a decision was made to proceed with diagnostic laparoscopy.

**Figure 3:**
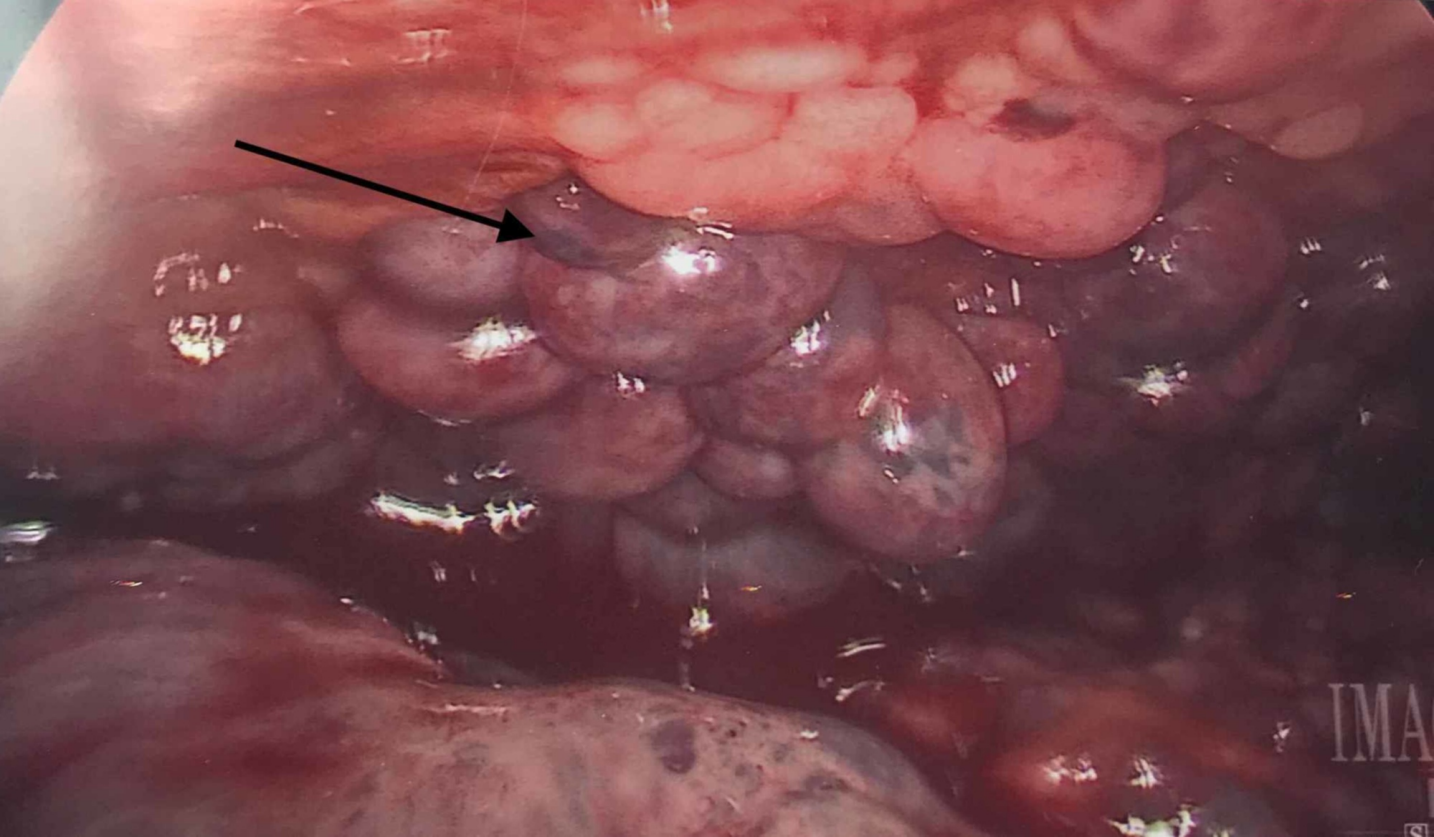
Intraoperative pictures showing intraabdominal purplish and friable metastatic deposits of melanoma (black arrow)

Intraoperatively, innumerable friable purple nodules were seen lining both visceral and parietal peritoneum in the intraabdominal cavity as well as the liver, spleen, omentum, and pelvic structures (Figure 3). There was no clearly identifiable source of bleeding observed except for diffuse oozing from the nodules. Due to the diffuse nature of the bleed, we were unable to achieve hemostasis via electrocoagulation or ligation. The absorbable hemostatic powder was placed over lesions that appeared to be oozing. Liver and omental biopsies were then obtained for frozen and permanent sections. Frozen sections showed a high-grade anaplastic tumor. A large Jackson Pratt drain was placed. We could not run the bowel due to the matted nature of the small bowel and the likelihood of exacerbating the bleed.

**Figure 4:**
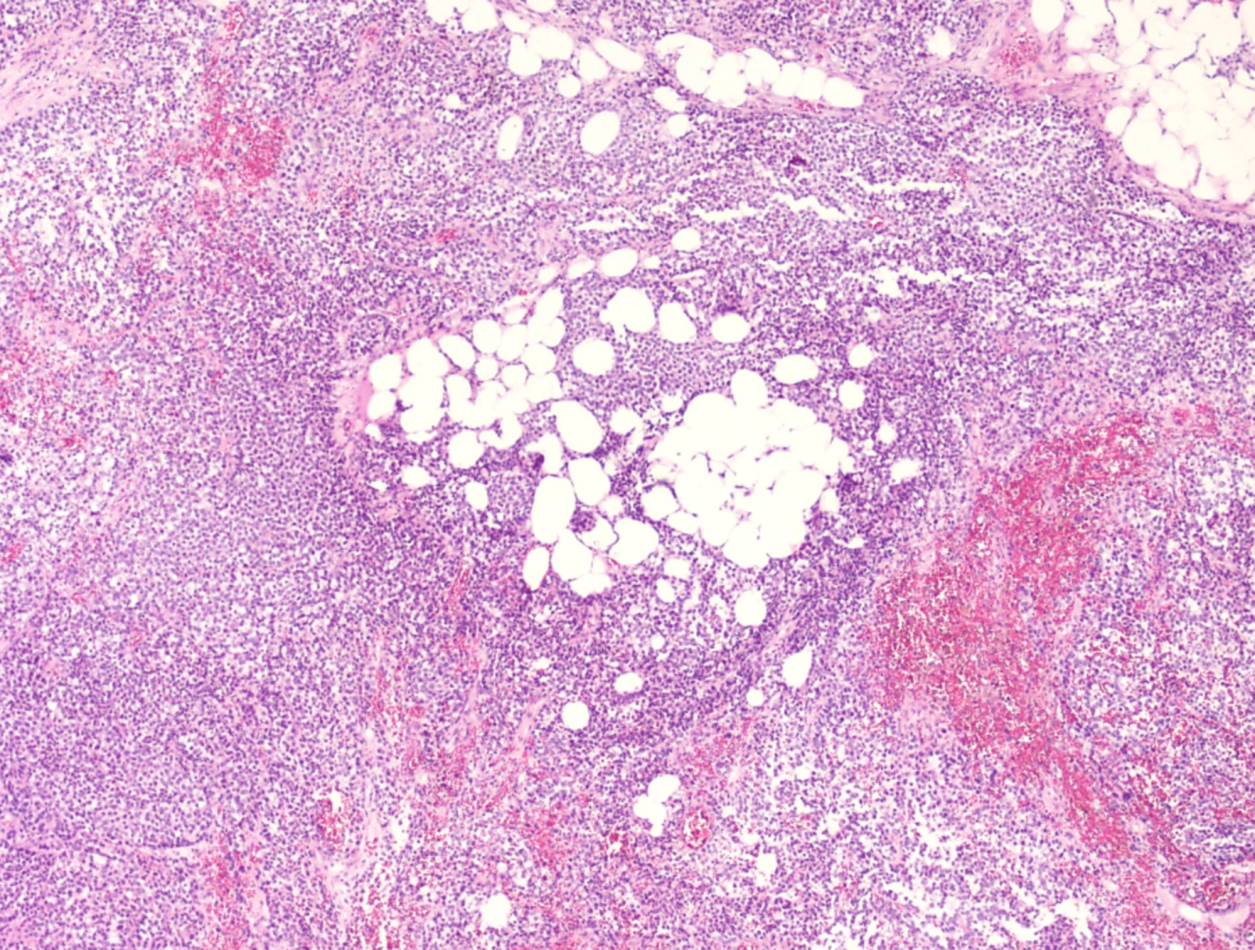
Microscopic slides showing H&E stains (100X) Low power view showing that the omentum is infiltrated by tumor cells.

**Figure 5:**
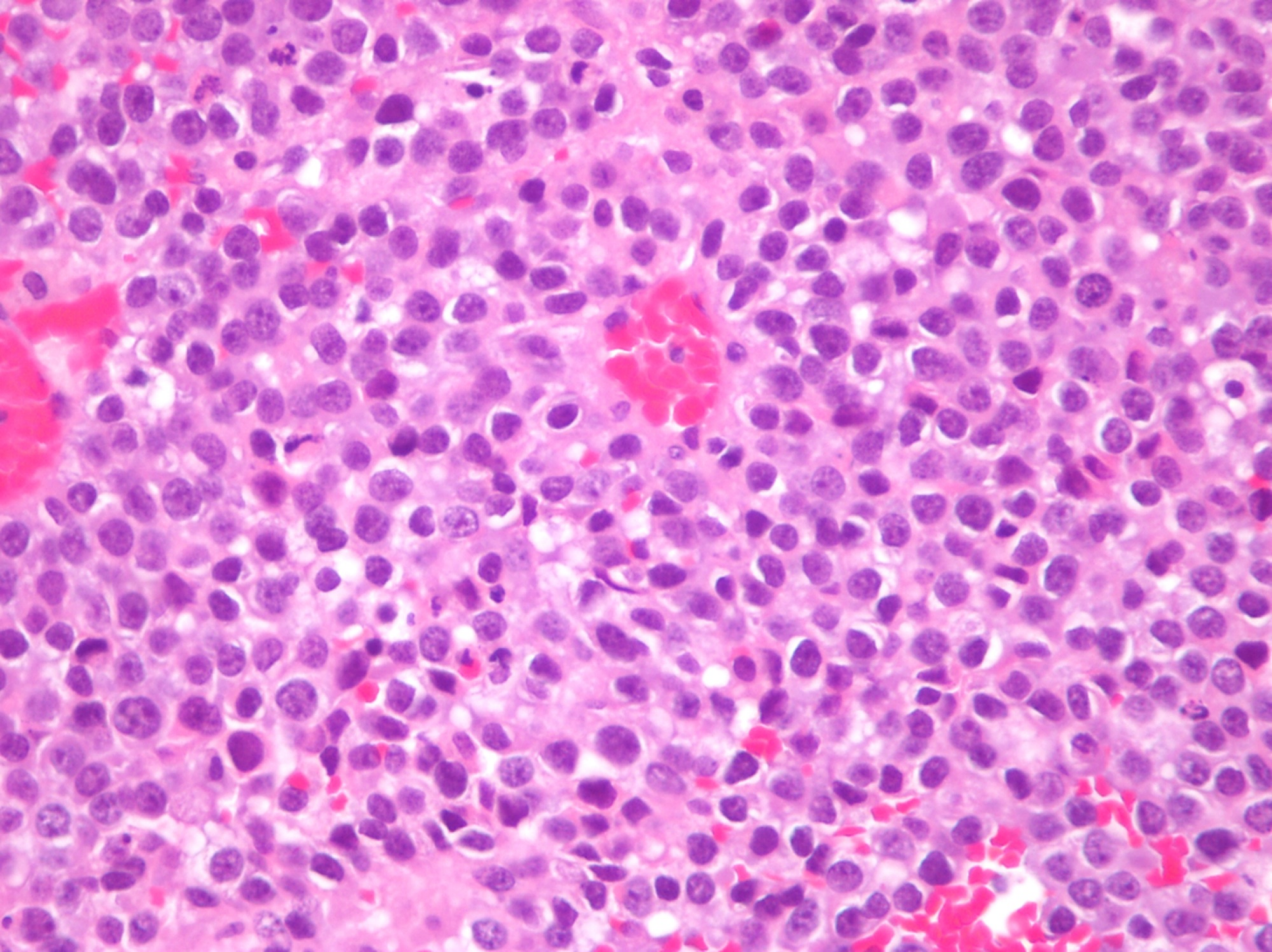
Microscopic slide showing H&E stains (400X) High power view shows small, round to oval malignant tumor cells with high nuclear-cytoplasmic ratio, punctate or inconspicuous nucleoli and frequent mitoses up to 100 per 10 high-powered field (HPF).

Postoperatively, she continued to have significant sanguineous output from the surgical drain. Her final pathology showed round malignant tumor cells, frequent mitoses up to 100 per 10 high power fields. Immunohistochemistry was positive for S100, HMB-45 and MelanA. Molecular studies showed neuroblastoma RAS (NRAS) consistent with melanoma (Figures 4 and 5). Microscopically, the omental tissue was infiltrated by small, round to oval malignant tumor cells with high nuclear-cytoplasmic ratio, punctate or inconspicuous nucleoli, and frequent mitoses up to 100 per 10 high power field. Immunohistochemical stains showed tumor cells positive for S100 (focally), HMB-45 (focally) and MelanA (diffusely). Immunohistochemical stains for CAM5.2, pankeratin, epithelial membrane antigen (EMA), CD45, desmin, smooth muscle actin (SMA), and CD99 were negative in the tumor cells. Molecular studies showed NRAS mutation, which was consistent with the diagnosis of malignant melanoma.

The patient and her family were informed of the poor prognosis of her condition, and options such as enrollment into a clinical trial and palliative care were discussed with the patient; however, they elected to be transferred to a tertiary center to pursue aggressive treatment options where she eventually passed away.

## Discussion

Malignant melanoma is a rare but serious form of skin cancer expected to affect 96,480 persons in the USA in 2019 [1]. Melanoma is aggressive and can metastasize to any organ system [3, 4]. Survival depends on the stage of disease at the time of diagnosis [3]. Early diagnosis with surgical excision can be curative [4]. The most common sites of metastasis are lung, liver, lymph nodes, brain, bone, and gastrointestinal (GI) tract. There are also less common reported metastases to the dura, eye, bile duct, peripheral nerves, uterus, vagina, rectum, and anus [6]. There are very few reported cases of peritoneal carcinomatosis [7]. Additionally, to our knowledge, there is no previous report of melanoma with peritoneal metastasis without a known primary, presenting with hemoperitoneum [6, 7].

The reported patient denied any history of skin cancer or any suspicious or new skin lesions. After the establishment of tissue diagnosis, we re-examined the patient for evidence of any cutaneous lesions; however, although we found none, her cutaneous exam was limited due to the patient’s inability to co-operate fully in the acute setting. For similar reasons, we were unable to perform aggressive workups, such as an ocular exam, proctoscopy, colonoscopy, or esophagogastroduodenoscopy (EGD) towards identifying the primary tumor. In the ideal setting, a thorough head to toe skin exams is needed, paying particular attention to areas where lesions are commonly missed such as the scalp, ears, axilla, umbilicus, nailbeds and interdigital areas. Examination of the mucosal surfaces such as the oral and vaginal mucosa is also important.

This patient’s presentation with nontraumatic hemoperitoneum is also rare. Most hemoperitoneum is secondary to nonmalignant gynecologic conditions. When complicated by malignant disease, hemoperitoneum can present be due to the rupture of liver tumors or other solid organ tumors [8].

The median survival of patients with melanoma with peritoneal metastasis is less than two months [7]. The general approach to the management of metastatic melanoma is multidisciplinary [4]. Surgery can be considered for the oligometastatic disease [2-4]. For advanced metastatic disease, the patient should be given the option to enroll in clinical trials [4]. There are ongoing melanoma clinical trials in the USA, aiming to find new and improved treatment options for patients with advanced disease. An example of a multicenter phase III randomized controlled trial (RCT), which is actively recruiting patients, is “A Study of NKTR-214 Combined With Nivolumab vs. Nivolumab Alone in Participants With Previously Untreated Inoperable or Metastatic Melanoma” [9]. Although this patient would have been a good candidate for this trial based on her clinical diagnosis, she may not qualify based on her decreased performance status per the Eastern Cooperative Oncology Group (ECOG) scale of performance status used by many clinical trials as an inclusion criterion [10].

## Conclusions

In conclusion, there is a rising incidence of malignant melanoma. Unusual presentation de novo or as a late recurrence can be expected. Clinicians should be aware of hemoperitoneum or peritoneal carcinomatosis as potential manifestations of malignant melanoma to expedite appropriate management.
